# MRI sets its sights on collagen

**DOI:** 10.7554/eLife.110375

**Published:** 2026-02-02

**Authors:** Fritz Schick

**Affiliations:** 1 Section of Experimental Radiology, Department of Diagnostic and Interventional Radiology, University Hospital of Tübingen Tübingen Germany; 2 German Center for Diabetes Research (DZD) Neuherberg Germany; 3 Institute for Diabetes Research and Metabolic Diseases of the Helmholtz Center, Munich at the University of Tübingen Tübingen Germany

**Keywords:** MRI, medical imaging, musculoskeletal system, collagen, Human

## Abstract

Reducing the echo time of a whole-body MRI scanner makes it possible to image collagen, an important structural protein found in bones and tendons.

**Related research article** van Schoor JD, Weiger M, Baadsvik EL, Pruessmann KP. 2026. Direct MRI of collagen. *eLife* **15**:RP109799. doi: 10.7554/eLife.109799.

MRI scanners enable doctors to diagnose a wide range of diseases – notably inflammatory, degenerative and tumorous diseases – without the need for harmful X-rays or radioactivity. They do this by measuring signals from the nuclei of hydrogen atoms in the body. These signals depend on the type of molecules the hydrogen atoms are bonded to, and the environment in which they are located, so they contain useful information about the body inside the MRI scanner.

It is not possible, however, to detect every hydrogen atom in a patient with MRI. To understand why this is so, we need to look at how the signals are generated. First, a strong magnetic field inside the MRI scanner forces the magnetic moments of all the hydrogen nuclei to generate a macroscopic magnetization. A short pulse of radio frequency radiation is then used to excite this nuclear magnetization, and the radiation subsequently emitted by the nuclei – which is called the echo signal – is recorded and used to construct an image (Brown et al., 2014; Dale et al., 2015). However, it is not possible to record an echo signal until the initial pulse of radiation has faded away. This time delay is called the echo time.

Echo signals decay exponentially with time, with some signals decaying faster than others. The echo signals from small mobile molecules, such as the water molecules found in all cells (or between cells) and the lipids found in fat cells, decay relatively slowly with time, so these signals are relatively easy to detect (Figure 1A).

**Figure 1.**
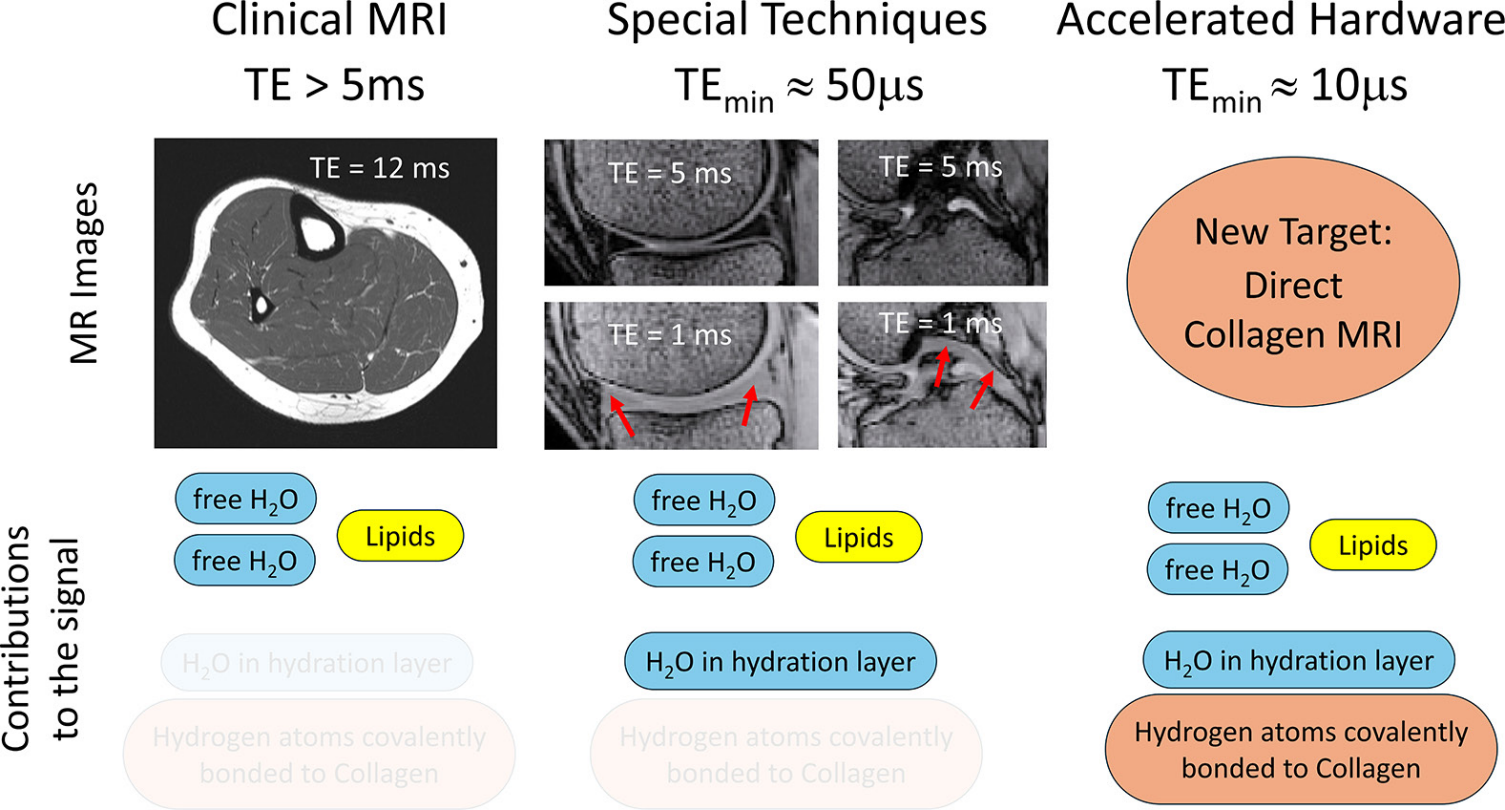
Reducing the echo time in MRI allows new features to be seen in images. Left: In clinical MRI, the echo time (TE) is typically between 5 and 150 milliseconds, so the only hydrogen signals that contribute to the image are those from small molecules that are mobile. This image of the lower leg was acquired with an echo time of 12 milliseconds. Centre: When the echo time is reduced to 1 millisecond, water molecules that are relatively immobile – such as those in the hydration layers around proteins such as collagen – contribute to the image. This means that structures such as menisci (red arrows, bottom left) and cruciate ligaments (red arrows, bottom right) show up clearly in images of the knee. These structures are not obvious in the images acquired with an echo time of 5 milliseconds (top). Right: van Schoor et al. were able to reduce the echo time to ~10 microseconds, which means that hydrogen atoms that are immobile – such as those bound covalently to collagen or other macromolecules – can contribute to MRI signals.

The shortest echo time that can be achieved with a typical MRI scanner in a clinical setting is about 5 milliseconds. This is too long to detect the signals from water molecules that are relatively immobile, such as those found in the hydration layers that surround macromolecules. However, the echo time can be reduced to about 50 microseconds by employing ‘special techniques’, such as using short radio frequency pulses for excitation (Robson et al., 2003; Garwood, 2013; Ma et al., 2022; Springer et al., 2014).

By enabling the detection of signals from relatively immobile water molecules in the hydration layer of macromolecules, these ultrashort echo times make it possible to visualise fibrocartilage, tendons and even water in bones (Figure 1B). However, water molecules that are directly bound to macromolecules remain invisible (Ma et al., 2016). A particular challenge for researchers working on MRI is to be able to image water molecules that are covalently bound to collagen, the protein that gives the musculoskeletal system its mechanical strength (Siadat and Ruberti, 2023).

Technically, it is difficult to reduce the echo time below about 50 microseconds because the radio frequency pulse used to excite the nuclei must decay completely before an echo signal can be recorded (Weiger and Pruessmann, 2019). Now, in eLife, Markus Weiger and colleagues at ETH Zurich and the University of Zurich – Jason Daniel van Schoor, Emily Louise Baadsvik and Klaas Paul Pruessmann – report that they have been able to reduce the echo time to just 10 microseconds in a whole-body MRI system (van Schoor et al., 2026). This required the development of highly sophisticated electronics that can rapidly dampen the oscillating circuit used for radio frequency excitation, thus allowing the MRI system to switch to recording mode very rapidly after excitation. In addition, they are able to very quickly manipulate how the magnetic field inside the scanner varies with position, which accelerates the spatial encoding of recorded signals and improves the quality of the images.

At first, samples of bovine tendons and bones, which have a high collagen content, were examined in a series of measurements. The samples were first imaged using an echo time of 10 microseconds, and then imaged again using progressively longer echo times. The signal decayed rapidly for times below about 40 microseconds, and decayed more slowly for longer times. The slow decay of the signal for longer times is consistent with previous measurements made using ultrashort time echoes, and these signals most likely originate from water molecules bound to collagen as a hydration layer in tendons, ligaments or menisci.

The samples were then treated to either remove water by freeze-drying, or to replace ordinary water with heavy water, which cannot produce an MRI signal in experiments like these (Ma et al., 2016), and the measurements at different echo times were repeated. Again, the researchers detected a signal that decayed rapidly at very short echo times (between 10 and 40 microseconds). Since the water molecules in the hydration layers around the collagen had been removed, the signal must have been coming from water molecules that were directly bound to collagen (Figure 1C).

A loss of collagen can lead to tears in tendons and ligaments, and possibly to bone fractures. Collagen is also a repair tissue that is stored in various functional tissues (including the lungs, liver and various muscles) for use during aging and/or in the event of injury. However, if the amount of collagen stored in these tissues becomes too high, it can lead to fibrosis (King et al., 2011). The ability to directly measure collagen signals should, therefore, open up new possibilities for MRI in diagnostics and therapy monitoring.
